# Human insulin/IGF-1 and familial longevity at middle age

**DOI:** 10.18632/aging.100071

**Published:** 2009-07-24

**Authors:** Maarten P. Rozing, Rudi G.J. Westendorp, Marijke Frölich, Anton J.M. de Craen, Marian Beekman, Bastiaan T. Heijmans, Simon P. Mooijaart, Gerard-Jan Blauw, P. Eline Slagboom, Diana van Heemst, on behalf of the Leiden Longevity Study (LLS) Group

**Affiliations:** ^1^ Department of Gerontology and Geriatrics, Leiden University Medical Center, 2300 RC, Leiden, the Netherlands; ^2^ Netherlands Consortium for Healthy Ageing (NCHA); ^3^ Department of Clinical Chemistry, Leiden University Medical Center, 2300 RC, Leiden, the Netherlands; ^4^ Department of Molecular Epidemiology, Leiden University Medical Center, 2300 RC Leiden, The Netherlands

**Keywords:** familial longevity, height, glucose handling, IGF-1, IGFBP3

## Abstract

Recently, we have shown that compared to controls, long-lived familial nonagenarians (mean age: 93.4 years) from the Leiden Longevity Study displayed a lower mortality rate, and their middle-aged offspring displayed a lower prevalence of cardio-metabolic diseases, including diabetes mellitus. The evolutionarily conserved insulin/IGF-1 signaling (IIS) pathway has been implicated in longevity in model organisms, but its relevance for human longevity has generated much controversy. Here, we show that compared to their partners, the offspring of familial nonagenarians displayed similar non-fasted serum levels of IGF-1, IGFBP3 and insulin but lower non-fasted serum levels of glucose, indicating that familial longevity is associated with differences in insulin sensitivity.

## Introduction

In Western societies, life expectancy has increased dramatically over the last century, but striking inter-individual differences in life expectancy remain [1]. Ample evidence has shown that healthy longevity is determined by a mix of genetic, environmental and chance elements. Because the odds of exceptional longevity runs in families, we designed the Leiden Longevity Study [2]. Recently, we have shown that the nonagenarian siblings included in the Leiden Longevity Study displayed a 41% lower risk of mortality compared to sporadic nonagenarians [3]. Moreover, compared to their partners, the offspring of nonagenarian siblings displayed a significantly lower prevalence of myocardial infarction,  hypertension and diabetes mellitus [3]. The differences in clinical phenotype observed after selection for familial longevity are in line with the lower prevalence of cardio-metabolic disease previously detected when offspring from sporadic centenarians were compared to offspring of parents who had died at average age [4] and when offspring from sporadic centenarians were compared to their partners [5]. Moreover, the observed lower mortality rate at high ages and better preservation of health at middle age indicates that resilience against disease and death may have similar underlying biological mechanisms that are influenced by genetic or familial factors.

Of the genetically determined pathways that have been implicated in longevity in a variety of different model organisms,  the evolutionarily conserved insulin/IGF-1 signaling (IIS) pathway clearly stands out in current literature (reviewed in [6]). Mutations in IIS components were first found to affect reproduction, metabolism, stress response and life span in *C. elegans* (reviewed in [7]). The link between reduced IIS signaling and longevity was subsequently also observed in *D. melanogaster*. Mutants in the *D*. *melanogaster* insulin receptor*InR *[8] and in the insulin receptor substrate *CHICO *[9] are both long-lived. Strikingly however, in both cases the long-lived phenotype was only observed for females. In addition to being long-lived, these *D. melanogaster* females are small, obese and infertile. In mice, selective disruption of the insulin receptor in the adipose tissue leads to a reduction in fat mass and extended longevity [10]. Increases in lifespan were also reported in mice with deletion of insulin receptor substrate 1 (IRS1) in whole body [11] or IRS2 only in the brain [12]. Moreover, dwarf mice exhibiting GH deficiency or resistance, including *Prop1^df/d^*[13], *Pit1^dw/dw^*[14], *GHRHR^lit/lit^*[14] and *GHR^-/-^*[15] all display hypoinsulinemia and enhanced insulin sensitivity along with extended longevity. In mice heterozygous for *igf1r *deletion (*Igf1r^+/-^*[16]) or containing a hypomorphic *igf1r* mutation (Midi mice [11]), only females, but not males, exhibited the long-lived phenotype.

Based on the similarities among the insulin/IGF-1 pathways in animals and humans, the possibility that modifications in the insulin/IGF-I signaling system could also extend lifespan in humans has been suggested. However, separating the roles of insulin and IGF-1 in mammals and their relevance for human healthy longevity has been difficult and generated much controversy. In humans, relatively low IGF-I levels have been associated with an increased risk of developing cardiovascular disease and diabetes, while relatively high IGF-I levels have been associated with an increased risk of developing cancer [17]. Moreover, in humans, an age-related decline in IGF-1 levels occurs [18], and at old age, low IGF-1 levels are associated with frailty [19], poor nutrition and cognitive decline [20] and an increased risk of death [21]. On the other hand, genetic variation in genes associated with down-regulation of the IIS pathway has been associated with human longevity in several instances, although, when moving up the evolutionary ladder, together with an increase in genome complexity, effect sizes became smaller [22]. Two studies have shown evidence for a role for genetic variation in the IIS pathway in body height as well as human longevity. First, earlier we found an association between genetic variation associated with reduced IIS pathway activity and shorter stature as well as improved old age survival in sporadic female octogenarians [23]. Second, offspring of sporadic female centenarians were shown to be smaller and display higher IGF-1 levels, indicative of IGF-1 insensitivity, while rare IGF-1R mutations associated with IGF-1 insensitivity were found enriched in centenarians [24]. Here, to investigate whether these results could be generalized to familial longevity, we have compared key anthropometric measures as well as serum parameters related to insulin/IGF-1 signaling in a group of middle-aged offspring of nonagenarian siblings and a control group of their partners of the Leiden Longevity Study.

## Results

### Metabolic characteristics of offspring compared to partners

Table 1 depicts the demographic and metabolic characteristics of the groups from the Leiden Longevity Study that were used for the present study. The group of offspring proportionately contained less diabetics than the group of partners (p = 0.001). After exclusion of diabetics, the group of offspring had lower non-fasted serum levels of glucose (p = 0.002) than the group of partners.  In addition, the group of offspring had a slightly more favorable lipid profile as compared to the group of partners.

### IGF-1/IGFBP3 and non-fasted glucose

Next we assessed whether the lower glucose levels observed among the group of offspring relative to thegroup of partners could be driven by differences in IGF-1 axis parameters.  Therefore we determined the association between serum IGF-1 / IGFBP3 molar ratios and non-fasted serum glucose levels. Higher ratios of IGF-1/ IGFBP3 were associated with lower serum glucose levels. One standard deviation increase in IGF-1/IGFBP3 ratio was associated with a decrease of 0.10 mmol/L serum glucose (SE: 0.05) among the group of partners (p = 0.05). The difference between partners and offspring in the change of glucose levels per standard deviation IGF-1/IGFBP3 ratio was not significant: 0.02 (SE: 0.06) nmol/L per year (p for interaction = 0.70).

### Measures of the IGF-1 axis in offspring compared to partners

Table 2 shows the comparison between the groups of offspring and partners for various IGF-1 axis parameters for males and females separately. In order to detect the effect of possible genetic differences in IGF-1 signaling between offspring and partners, we also determined anthropometrical characteristics in subjects of both study groups (Table 2). With regard to serum IGF-1 axis parameters, no differences were observed between the group of offspring and the group of partners in both sexes. Likewise, the study groups showed no differences in terms of sex-specific body stature, i.e. height, weight and body mass index.

**Figure 1.**
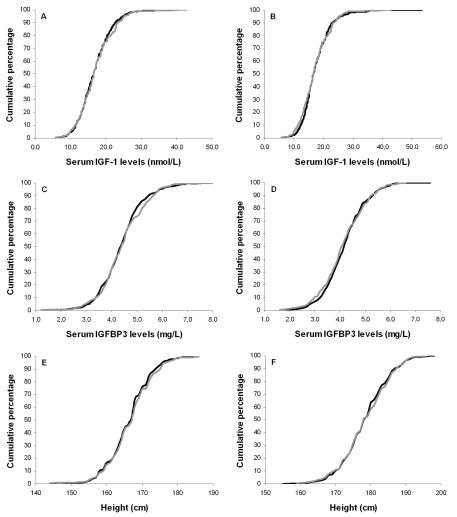
Cumulative distribution curves of serum IGF-1 levels, serum IGFBP3 levels and height. Cumulative distribution curves of IGF-1 levels for offspring and partners among females (**A**) and males (**B**); Cumulative distribution curves of IGFBP3 levels for offspring and partners among females (**C**) and males (**D**); Cumulative distribution curves of height for offspring and partners among females (**E**) and males (**F**). Black lines represent offspring, gray lines represent partners.

Next, we determined whether the distribution of serum IGF-1 axis parameters and anthropometrical parameters were different between offspring and partners.  Figure 1 displays the cumulative distributions of IGF-1, IGFBP3 and height among partners and offspring for both sexes separately. No differences in height were observed between offspring and partners in the tails of the IGF-1 and IGFBP3 distribution curves. Taken together, the cumulative distribution curves do not suggest enrichment of high or low IGF-1 axis parameters nor large or short statures among the groups of offspring versus partners.

**Table 1.** Comparison of demographics and serum parameters between offspring and partners for males and females combined. *Age is presented as median with interquartile range. Serum parameters are presented as mean values with 95% confidence intervals.  Mean values, 95% confidence intervals and p-values were calculated using a linear regression model, adjusted for age and sex. LDL denotes low-density lipoprotein and HDL high-density lipoprotein. **Data are presented as geometric means with 95% confidence intervals. † Mean values, standard error of the mean and p-value for Total cholesterol, LDL cholesterol, HDL cholesterol, , Triglycerides and Free Fatty Acids were adjusted for lipid lowering agents (fibrates, niacin, bile acid sequestrants, 3-hydroxy-3-methylglutaryl-coenzyme A reductase inhibitors).

	Offspring	Partners	p-value	
**Demographics**				
Participants - n	1171	542		
Diabetics - n (%)	46 (3.9)	42 (7.7)	**0.001**	
Females - n (%)	633 (54.1)	302 (55.7)	0.57	
Age (year)	59.2 (55.0 - 64.1)	58.8 (54.3 - 63.7)	0.15	
			
**Serum parameters (non-diabetics) **				
Participants - n	1125	500	
Glucose (mmol/L)	5.69 (5.62 - 5.76)	5.87 (5.76 - 5.97)	**0.002**
Insulin (mU/L )**	14.4 (13.6 - 15.4)	15.4 (14.0 -16.8)	0.21
			
Total cholesterol (mmol/L)†	5.56 (5.47 - 5.65)	5.62 (5.52 - 5.72)	0.40
LDL cholesterol (mmol/L)†	3.32 (3.24 - 3.39)	3.37 (3.29 - 3.45)	0.33
HDL cholesterol (mmol/L)†	1.46 (1.42 - 1.49)	1.43 (1.39 - 1.47)	0.24
Triglycerides (mmol/L) **,†	1.50 (1.44 - 1.55)	1.57 (1.50 - 1.65)	0.09
Free fatty acids (mmol/L) **,†	0.27 (0.26 - 0.28)	0.27 (0.26 - 0.29)	0.38


**Figure 2.**
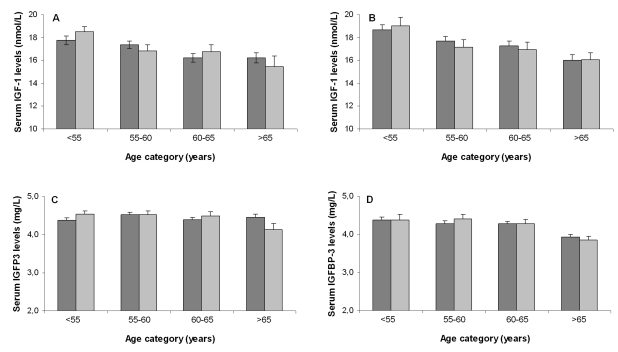
Association between age categories and serum IGF-1 levels for offspring and partners among females (**A**) and males (**B**) and association between age categories and serum IGFBP3 levels for offspring and partners among females (**C**) and males (**D**). Dark bars represent offspring, light bars represent partners. Number of participants per age category for females (offspring/ partners): category <55: 156/110; category 55-60: 194/83; category 60-65: 146/66; category >65: 114/27.  Number of participants per age category for males (offspring/ partners): category <55: 133/42; category 55-60: 140/49; category 60-65: 140/57; category >65: 102/66.

IGF-1 levels have been consistently reported to progressively decline with age. To determine whether this observation applied to the groups that were used in the present study, we assessed the association between serum IGF-1 levels and serum IGFBP3 levels with age. Figure 2 displays the sex-specific serum IGF-1 and IGFBP3 levels for different age categories among offspring and partners.  Serum IGF-1 levels declined with age in both female partners (-0.14 (SE: 0.04) nmol peryear increase; p<0.001) and male partners (-0.16 (SE: 0.05) nmol/L per year increase; p =0.001). The difference in annual change in serum IGF-1 levels between partners and offspring was not significant: 0.01 (SE: 0.05) nmol/L per year (p for interaction= 0.79) for females and 0.01 nmol/L (SE: 0.06) per year (p for interaction=0.83) for males. Similarly, no differences between partners and offspring were observed in terms of annual change in serum IGFBP3 levels: 0.01 mg/L (SE: 0.01) (p for interaction = 0.47) for females and 0.02 mg/L (SE: 0.01) (p for interaction =0.10) for males.

**Table 2.** Comparison of anthropometrics and IGF-1 axis parameters between offspring and partners for females and males separately. Data are presented as means with 95% confidence intervals. All analyses were adjusted for age. *Diabetic subjects were excluded from analyses.

	Offspring*	Partners*	p-value
**Females (n)**	610	286	
IGF-1 axis serum parameters			
IGF-1 (nmol/L)	17.1 (16.7 - 17.5)	17.1 (16.5 - 17.7)	0.99
IFGBP3 (mg/L)	4.44 (4.36 - 4.53)	4.47 (4.36 - 4.57)	0.72
IGF-1/ IGFBP3 (molar ratio)	0.11 (0.11 - 0.11))	0.11 (0.11 - 0.11)	0.60
			
Anthropometrics			
Height (m)	166.8 (166.2 - 167.3)	166.9 (166.1 - 167.7)	0.79
Weight (kg)	69.2 (68.2 - 70.3)	70.2 (68.9 - 71.6)	0.25
Body Mass Index (kg/m^2^)	24.9 (24.5 - 25.3)	25.2 (24.8 - 25.7)	0.25
			
**Males (n)**	515	214	
IGF-1 axis serum parameters			
IGF-1 (nmol/L)	17.5 (17.0 - 17.9)	17.3 (16.6 - 18.0)	0.75
IGFBP3 (mg/L)	4.22 (4.13 - 4.30)	4.20 (4.08 - 4.32)	0.85
IGF-1/ IGFBP3 (molar ratio)	0.12 (0.12 - 0.12)	0.12 (0.12 - 0.12)	0.82
			
Anthropometrics			
Height (m)	178.7 (178.1 - 179. 4)	179.1 (178.2 - 180.0)	0.44
Weight (kg)	82.0 (80.9 - 83.0)	82.4 (80.8 - 84.1)	0.61
Body Mass Index (kg/m^2^)	25.6 (25.4 - 25.9)	25.7 (25.2 - 26.1)	0.96

## Discussion

The main findings of this study are twofold. First, consistent with the lower prevalence of diabetes observed earlier, non-fasted serum glucose levels were lower in the offspring of familial nonagenarians when compared to their partners. Second, we did not observe differences in non-fasted serum levels of IGF-1, IGFBP3 or in height between the groups of offspring and partners, nor in the rate of the decline of levels of IGF-1 or IGFBP3 over chronological age. Taken together,  these data indicate that familial longevity is associated with differences in glucose handling, which are not explained by major differences in IGF-1 and/or IGFBP3 levels.

The link between reduced IIS activity and longevity is evolutionarily conserved from worms to rodents, with effects on longevity often being stronger in the female sex. However, separating the roles of insulin and IGF-1 in mammals has been very difficult and generated much controversy. Because the actions of GH, insulin and IGF-1 are largely interwoven, genetic modification of the GH/IGF-1 axis in mammals also entails differences in the regulation of glucose metabolism. Interestingly, the hallmark phenotype of all long-lived mouse models containing mutations that induce GH/IGF-1 deficiency or resistance, is their enhanced insulin sensitivity [6]. Previously, we observed a lower prevalence of diabetes in the offspring group [3]. Here, we show that after exclusion of all diabetics, lower non-fasted glucose levels were observed in the group of offspring of familial nonagenarians as compared to the partners. The lower non-fasted glucose levels in offspring compared to partners are suggestive of a better glucose handling and/or higher insulin sensitivity in familial longevity, which is in line with the hallmark phenotype observed in the many long-lived mammalian IIS mutants. Other data also support a link between preserved insulin sensitivity and human longevity. While insulin sensitivity generally declines with age in humans [25], sporadic long-lived centenarians have been shown to exhibit an exquisite insulin sensitivity, comparable to that of young adults [26].

The preserved insulin sensitivity observed in centenarians, co-occurred with relatively high levels of IGF-1/IGFBP3, which has lead to the suggestion of causal link between the preserved insulin sensitivity and levels of IGF-I/IGFBP3 [27]. In rats, IGF-1 and IGFBP3 were shown to have opposing (centrally mediated) effects on glucose metabolism, with IGF-1 acting as an insulin sensitizer, and IGFBP3 as an insulin inhibitor [28]. Similarly, in humans, IGF-1 administration was found to increase glucose uptake and inhibit hepatic glucose production in healthy subjects [29], and low serum IGF-1 levels were found associated with glucose intolerance [30]. In line with these findings, we also observed a negative association between IGF-1/IGFBP3 levels and non-fasted glucose levels in both our study groups, but neither this association nor the mean levels of IGF-1 and IGFBP3 were different between the offspring and partner groups. Our observation of improved glucose handling in the absence of major differences in IGF-1/IGFBP3 levels resembles  the Effects observed upon caloric restriction in humans. In contrast to model organisms, in humans, IGF-1 levels were not found to be decreased upon caloric restriction, while insulin sensitivity was increased upon caloric restriction in humans as in model organisms [31]. The lack of differences in BMI, as well as preliminary data on food intake, indicate however that the observed difference in glucose handling between the groups of offspring and partners can not be explained by a lower caloric intake in the offspring group.

The observation of improved glucose handling in the absence of major differences in IGF-1/IGFBP3 in familial longevity does not rule out the possibility that genetic variations affecting IGF-1/IGFBP3 levels do contribute to human longevity. Recently, it was shown that centenarians exhibited a relative enrichment for rare genetic variants in the IGF-1 receptor which resulted in high levels of IGF-1/IGFBP3 coexisting with low levels of IGF-1 signaling [24]. Also, earlier we and others showed that common genetic variations affecting IGF-1 signaling might contribute to differences in mortality in the population at large [23,32], but the phenotypic effects associated with such variants (smaller stature, differences in serum levels of IGF-1 and/or IGFBP3) do not form a distinctive part of the hallmark phenotype of preserved glucose handling which we found associated with familial longevity.

## Methods

Leiden Longevity Study. In the Leiden Longevity Study, 420 families were recruited consisting of long-lived Caucasian siblings together with their offspring and the partners thereof [2,23]. Families were recruited if at least two long-lived siblings were alive and fulfilled the age-criterion of 89 years or older for males and 91 year or older for females. There were no selection criteria on health or demographic characteristics. For 2465 of the offspring and their partners, non-fasted serum samples taken at baseline were available for the determination of endocrine and metabolic parameters. Between November 2006 and May 2008, for 2235 of the offspring and their partners, information on medical history was obtained from the participants' general practitioner (response: 91%). For 2255 of the offspring and their partners, information on the use of medication was obtained from the participants' pharmacy (response: 92%). For 2184 of the offspring and partners a general questionnaire containing information on lifestyle and self-reported height and weight was obtained (response: 89%). For the present study, for a total of 1713 of the offspring and their partners, serum as well as information on medical history on diabetes and information on medication use and the general questionnaire were available (inclusion: 70%). After exclusion of subjects with diabetes in medical history (n=87) and/or non-fasted glucose lower than 11 mmol/L (n=1) and/or use of glucose lowering medication (n=37), a sample of 1625 subjects was available for the current study. The Medical Ethical Committee of the Leiden University Medical Centre approved the study and informed consent was obtained from all subjects.

Biochemical analysis . All serum measurements were performed with fully automated equipment. For insulin‑like growth factor-1 (IGF-1), insulin-like growth factor binding protein 3 (IGFBP3) and insulin, the Immulite 2500 from DPC (Los Angeles, CA, USA) was applied. CVs for these measurements were all below 8%. For glucose, total cholesterol, HDL-cholesterol, triglycerides, free fatty acids (FFA) the Hitachi Modular or the Cobas Integra 800, both from Roche, Almere, the Netherlands were applied. CVs of these measurements were all below 5 %.

Medication use *.* Lipid lowering agents were defined as fibrates, niacin, bile acid sequestrants, 3-hydroxy-3-methylglutaryl-coenzyme Areductase inhibitors (ATC code C10).

Calculations and statistical analysis. For estimation of the level of LDL cholesterol the Friedewald formula was applied (LDL cholesterol [mmol/l] = total cholesterol - HDL cholesterol - [triglycerides/2.2]), whereby participants with a triglyceride concentration higher than 443 mg/dl (5 mmol/l) were excluded. For molar comparisons between IGF-1 and IGFBP3, the following molecular masses were used in the calculation: IGF-1: 7.5 kDa and IGFBP3: 28.5 kDa.

Distributions of continuous variables were examined for normality and logarithmically transformed, when appropriate and used in all calculations. Geometric means (with 95% confidence intervals (CI)) are reported for transformed variables (insulin, triglycerides and free fatty acids). All differences in mean serum levels and anthropometrics between the groups of offspring and partners were assessed with the use of linear regression, adjusted for sex, age and correlation of sibling data using robust standard errors in STATA. The relation between IGF-1/IGFBP3 molar ratio (expressed in Z-scores and restricted to values within 3 standard deviations (SDs) from the mean) and glucose was assessed with the use of a linear mixed model, adjusted for sex, age and correlation of sibling data in SPSS. The cumulative distributions of IGF-1, IGFBP3 and height were calculated in SPSS. The change in levels of IGF-1, IGFBP3 over chronological age as a continuous variable was assessed with the use of a linear mixed model, adjusted for age and correlation of sibling data in SPSS. The Statistical Package for the Social Sciences (SPSS) program for Windows, version 14.0, and STATA version 10.0 were used for data analysis, and plots were drawn in Excel.
